# Hepatitis Delta: Epidemiology, Diagnosis and Management 36 Years After Discovery

**DOI:** 10.1007/s11894-013-0365-x

**Published:** 2013-11-30

**Authors:** Mazen Noureddin, Robert Gish

**Affiliations:** 1 Division of Gastroenterology and Hepatology, Keck School of Medicine, University of Southern California, 2011 Zonal Avenue, HMR 101, Los Angeles, CA 90033 USA; 2 Robert G. Gish Consultants, LLC, San Diego, CA USA; 3 St. Joseph’s Hospital and Medical Center, Phoenix, AZ USA; 4 University of Nevada, Las Vegas, 6022 La Jolla Mesa Drive, San Diego, CA 92037 USA

**Keywords:** Hepatitis, RNA, Chronic hepatitis, Hepatitis delta virus, Cirrhosis, Interferon-alpha

## Abstract

With recent studies showing increased prevalence of hepatitis delta (HDV) even in the US, Australia, and some countries in Europe, and very high prevalence in endemic regions, HDV infection is far from being a disappearing disease. Although immigrants from endemic countries have been shown to have increased risk, studies have clearly shown that the disease is not solely appearing in traditional high-risk groups. Recent studies provide increasing evidence that sexual transmission may be an important factor in HDV infection spread. Based on the totality of evidence showing increased disease progression and substantially increased risk of cirrhosis in HDV-infected CHB patients, and the current studies showing higher than expected prevalence, it is time to call for HDV screening of all CHB patients. HDV viral load detection and measurement should be considered in all patients whether or not they are anti-HDV-positive. With universal screening of CHB patients for HDV, earlier diagnosis and consideration of treatment would be possible. Current treatment of HDV is IFN-based therapy with or without HBV antivirals, but current research indicates the possibility that prenylation inhibitors, entry inhibitors, HBsAg release inhibitors, or other therapies currently in the pipeline may provide more effective therapy in the future. In addition, universal screening would serve the important public health goal of allowing patients to be educated on their status and on the need for HDV-negative patients to protect themselves against superinfection and for HDV-infected patients to protect against transmission to others. Further studies and global awareness of HDV infection are needed.

## Introduction

Hepatitis delta virus (HDV) was first discovered in 1977 by Mario Rizzetto in Turin, Italy [1]. Collaboration between Turin and US scientists on experiments in chimps demonstrated that the delta antigen requires HBV for its ability to infect hepatocytes [2–4]. HDV is the smallest human RNA virus with a small circular RNA genome of approximately 1,700 bases; the genome is single-stranded negative sense and forms a covalently closed circle [5]. Because of a large amount of base pairing, the viral RNA takes on a rod-like structure. The RNA then encodes a protein called the delta antigen, which is subsequently encased in an envelope embedded with hepatitis B surface antigen (HBsAg) [5]. HDV infection is significant because, although it suppresses hepatitis B virus (HBV) replication, it can cause severe liver disease that may include fulminant liver failure and rapid progression to cirrhosis and hepatic decompensation, as well as an increased risk of liver cancer. Since HDV can only cause infection in the presence of HBV, it was thought that the widespread introduction of HBV vaccine would ultimately result in decreased prevalence of HDV. However, current studies have shown that ongoing high prevalence remains in many parts of the world.

## Prevalence

It was long ago established that HDV is found throughout the world, with higher prevalence in countries with populations of low socioeconomic status in Africa and South America, as well as in Turkey, Mongolia, southern Italy, and the Soviet Union [6]. HDV antibodies were found in up to 30 % of chronic hepatitis B (CHB) patients in some of these countries [7]. On the other hand, there was a lower prevalence in CHB patients in northern Europe and North America, with HDV infection thought to be mainly restricted to intravenous drug users [4, 7]. In the second decade after discovery, there was a decrease in prevalence of HDV which was thought to be mainly the result of the implementation of the HBV vaccine [8]. This led to decreased awareness and testing for HDV which further contributed to the perception that the virus was on its way to being eradicated [8]. Unfortunately, recent studies have shown the contrary.

In the United States, a recent study found that 8 % of CHB patients in northern California were coinfected with HDV [9••]. Interestingly, 73 % of the coinfected patients had cirrhosis compared to 17 % of patients who were only HBV infected. In addition, 63 % of these patients were born in North America and only 26 % reported past IV drug use, showing that the disease is not solely appearing in immigrants and drug users. These findings clearly emphasize the importance of HDV screening of all CHB patients, not just those with established risk factors or cirrhosis. However, drug use is clearly still a major risk factor, with a study from Baltimore showing 50 % HDV coinfection in CHB patients who are IV drug users [10].

Worldwide, it is estimated that 15–20 million people are HDV infected, with widely varying prevalence, depending on the region [7, 11]. The highest prevalence is seen in the Mediterranean basin, the Middle East, central and northern Asia, western and central Africa, the Amazonian basin (Brazil, Peru, Venezuela, and Colombia), the Pacific islands [12], and Vietnam [13]. In a recent study in the western Brazilian Amazon region, 41.9 % of HBsAg-positive patients were found to be coinfected with HDV, with prevalence over 60 % among individuals aged 20–39 years [14]. The higher prevalence in this age group suggests the likelihood of sexual transmission. In a 2013 study from West Africa (Mauritania), it was found that up to 30 % of CHB patients had anti-HDV antibodies [15]. Among these, 62.2 % were HDV-RNA-positive. A recent study from the Middle East showed that, in Zahedan, Iran, 17 % of CHB patients were positive for anti-HDV antibodies [16]. A recent meta-analysis showed that 14.8 % of asymptomatic HBsAg-positive patients in the eastern Mediterranean region are coinfected with HDV and that cirrhosis is common in this group [17]. A recent study in Vietnam showed HDV prevalence of 10.7 % in CHB patients [13], a prevalence much higher than expected since previous studies had shown either very low levels of HDV infection in Vietnamese CHB patients [18] or none at all [19]. These studies confirm the continuing high prevalence of HDV infection in the Amazonian basin, the Middle East, the eastern Mediterranean region, and western Africa, and show the unexpectedly high prevalence in Vietnam. At the other end of the prevalence scale, a recent report from Egypt showed that 4.7 % of CHB patients are coinfected with HDV [20]. In a study in India which assessed 450 CHB patients for coinfection with HIV or HDV, it was found that 4.8 % were HBV/HDV-coinfected, while only 1.5 % were HIV/HBV-coinfected and 0.8 % were HBV/HDV/HIV-tri-infected [21]. However, HBV/HDV coinfection was substantially higher (45.8 %) in patients aged 21–40 years old. Again, the higher prevalence in this age group suggests that the transmission of HDV could be due to sexual transmission.

In Europe, where HDV had been thought to be decreasing, studies in 2013 showed a trend toward increasing prevalence. In a large prospective Greek study which has followed 4,673 CHB patients from 1997 to the present, of the 2,137 patients who have been screened for HDV, 4.2 % were found to be anti-HDV-positive, with substantially greater prevalence seen in immigrants (7.5 %) than in native Greeks (2.8 %). The study showed that HDV testing decreased substantially over time, dropping from 57 % prior to 2003 to only 35.3 % thereafter [22]. Within 2.3 years of follow-up, new HDV infection occurred in 2.2 adults and 8.7 children per 1,000 HBsAg-positive patients annually. A study from Belgium showed that 5.5 % of CHB patients are HDV-coinfected, a prevalence that is higher than previously reported [23]. In London, the prevalence of HDV infection was found to be 2 % among CHB patients [24]. The majority of HDV-infected patients were either IV drug users or immigrants. In comparison, other European countries were found to have substantially higher prevalence in some populations, with coinfection reported in 20.4 % of CHB patients in Bucharest, Romania, and 20.9 % of 1,220 CHB immigrants from Equatorial Guinea living in Spain [25, 26].

Finally, an important Australian study found that of the 2,314 Victoria residents tested for HDV infection from 2000 to 2009, 110 (4.75 %) were found to be HDV-infected [27]. Both the number of people testing positive and the number of tests conducted steadily increased between 2005 and 2009. The majority of cases were immigrants (71.4 %) and male (77 %). The investigators concluded that their findings emphasize the need for routine HDV testing of all CHB patients. We agree, and believe that, since HDV coinfection results in worse disease, including cirrhosis at younger age, the continuing high prevalence in many countries, and trend toward increasing prevalence in some parts of the world, these clearly point to the need to revise screening guidelines to call for HDV screening in all CHB patients. With multiple studies showing particularly high prevalence in immigrants from endemic countries, they should be considered as being at particularly high risk of being infected.

## Clinical Course

The transmission of HDV can occur sexually (via semen or vaginal secretions), through blood (needle stick injuries, injection drug use, and transfusions), and perinatally. Recent studies provide increasing evidence that sexual transmission may be a more important factor in the spread of the disease than has previously been appreciated. The studies discussed above that found substantially higher prevalence of HDV coinfection in patients aged 20–39 years (60 % vs. an overall prevalence of 41.9 %) [14] and in 21–40 year olds (45.8 % vs. an overall prevalence of 4.8 %) [21] highlight the importance of sexual transmission and point to the need to first screen patients and then educate HDV-negative patients about the need for protection against superinfection and HDV-infected patients about the need to protect against transmission to others.

HDV can be transmitted only in the existence of concomitant HBV infection as one of two patterns, simultaneous HBV/HDV infection (coinfection) or HDV infection of an individual already chronically infected with HBV (superinfection). The coinfection pattern ranges from mild to severe or fulminant hepatitis [28]. Coinfection is usually self-limited, with 20 % of cases progressing to cirrhosis. However, coinfection can lead to more severe fulminant hepatitis compared to superinfection [29]. Superinfection may present as exacerbation of disease in previously asymptomatic CHB patients, worsening of disease in CHB patients, or as acute hepatitis [29]. Superinfection is usually confirmed with positive HDV antibodies and negative IgM anti-HBc [29]. Chronic hepatitis D is acquired in 90 % of superinfection cases and leads to cirrhosis in 5–10 years in 70 % of the patients. Cirrhosis incidence is three times higher with HDV infection than with HBV monoinfection [29]. Cirrhosis may continue for many years, but a high percentage of patients will decompensate, develop hepatocellular carcinoma (HCC), and/or receive liver transplantation [30]. HCC association with chronic HDV is still controversial, with some studies showing up to a three-fold increase in risk and others showing no increase [30]. Pathogenicity can influence outcome in HDV infection and may vary between genotypes [31]. At least 8 genotypes of HDV have been described. Genotype I has been found in the United States, Europe, the Middle East, North America, and North Africa. Genotype II has been found in the Far East, Genotype III in South America, and Genotypes IV–VIII in Africa [31].

## Diagnosis

With the high prevalence in many countries already discussed, including the surprisingly high prevalence in the US despite its longstanding HBV vaccination programs, and the trends toward increasing prevalence in some parts of the world, it seems more than reasonable to advocate for all CHB patients to be screened for HDV infection. With multiple studies showing particularly high prevalence in immigrants from endemic countries, they should be considered as being at particularly high risk of being infected, and consideration should be given to the development of programs to reach this population. There should absolutely be testing of all members of high-risk groups, including injection drug users, men who have sex with men, hemodialysis patients, anyone who has sexual contact with confirmed HDV-infected persons, and health-care and public safety workers. Patients with chronic HBV who experience clinical deterioration should also be tested [32].

HDV antigen can be measured; however, it is only transiently detectable in serum. The detection of HDV antibodies in HBsAg-positive patients is usually the initial step in the diagnosis of HDV; however, these antibodies can be falsely negative. Recently, there has been an emphasis on testing for HDV RNA viral load as more laboratories have gained experience in measuring it. To date, this method has mostly been done in academic centers.

It is not yet commercially available in the US, although the PCR test is becoming more available in some other countries [33•, 34]. In the US, a one-step TaqMan quantitative reverse transcription polymerase chain reaction (qRT-PCR) assay has been developed for detection of HDV RNA, which has been found to be highly reliable and accurate in detecting viral load [35]. It is likely that viral load testing will be available soon in the US in commercial laboratories, and, in the meantime, all efforts should be made to send samples, especially with suspect populations, to laboratories experienced in measuring viral load. Any physician or associated medical personnel can submit specimens to the CDC for HDV testing if the person is suspected of having HDV. The specimens should be packaged in accordance with the CDC requirements for shipping biological specimens (http://www.cdc.gov/hepatitis/HEV/PDFs/HRL_PCRSampleHndlgShpg_20080615.pdf) and must be accompanied by the form from this link completed in its entirety: http://www.cdc.gov/hepatitis/HDV/index.htm.

In HDV-positive patients, it is critical to distinguish acute HDV and HBV infection (coinfection) from HDV superinfection in CHB patients, since the prognosis and management are different. Acute infection with HDV concomitant with acute infection with HBV usually appears first as IgM anti-HDV and then converts to IgG anti-HDV; HDV RNA levels reach high levels in the serum. HBV IgM anti-HBc will also be found to be positive in this acute HDV/HBV coinfection. In superinfection, HDV antibodies appear early as IgM, followed by IgG anti-HDV, whereas anti-HBc is IgG only. Both HDV and HBV antibodies may increase in superinfection as the HDV disease progresses to chronicity, and may be present in high titers along with positive HDV RNA. Finally, a Baseline Event-Anticipation (BEA) score has been developed to predict the risk of development of liver-related complications (decompensation, hepatocellular carcinoma, liver transplantation, and/or death) in chronic HDV patients [36•]. This easy to apply clinical instrument uses age, gender, region of origin, INR, platelet number, and bilirubin to calculate the score. Factors associated with a poor long-term outcome include age above 40, male sex, low platelet counts, high bilirubin, elevated INR, and southeast Mediterranean origin.

## Treatment

To date, the unique HDV replication cycle and its association with HBV have led to difficulties in treatment regimens and inadequate response rates. The ultimate approach for eradication of HDV is the clearance of HBsAg, not just sustained HDV virological response (negative HDV RNA 6 months after stopping treatment) [37]. If HBsAg remains positive, HDV remains infectious, even if the HBV or HDV viral titers are low. This has led to the concept that eradication of HBsAg might also clear HDV infection. However, despite inhibition of HBV replication and, in some cases, HBsAg clearance, the clearance of HDV has not been seen when oral antivirals against HBV were used alone. Lamivudine, ribavirin, famciclovir, adefovir, and entecavir have shown little or no effect when used alone to treat HDV, regardless of the duration of treatment [38–42].

Interferon-alpha is the only therapy that has been shown to be at least somewhat effective for treatment of HDV (Table 1), leading to virological, biochemical, and histological response in some patients. Weekly injection of PEG-IFN-a is currently used for 12–18 months. A 2013 review concluded that recent trials have shown HDV SVRs in 25–30 % of patients treated with pegylated interferons (Peg-IFN) [43]. However, in one of the few trials with truly long-term follow-up, Heidrich and colleagues found that even in patients who are HDV-RNA-negative 6 months after Peg-IFN-based treatment, late relapses may occur [44••]. In their initial study, Peg-IFN alfa-2a treatment for 48 weeks, with or without adefovir, resulted in 28 % of the patients having undetectable HDV-RNA 6 months post-treatment [42]. Long-term follow-up data were available for 37 of the 60 patients treated with Peg-IFN, with a median time of follow-up of approximately 4 years. Of the 16 patients entering the follow-up study who were HDV-RNA-negative 6 months after treatment, half (8 individuals) tested HDV-RNA-positive at least once during further follow-up, leading the investigators to call for close monitoring of patients post-Peg-IFN therapy, even in patients who are HDV-RNA-negative 6 months after therapy completion [44••].

**Table 1:** Outcomes of interferon therapy

Study	Type	*n*	Treatment regimen	Duration	Treatment response definition	Treatment outcome
Farci et al. [45]	Randomized	42	IFN a-2a (9M) (14) vs. IFN alpha-2a (3 M) (14) vs. No therapy (14)	48 weeks	At EOT: **Biochemical** (ALT) **Virological** (HDV RNA eradication) **Complete** (ALT + RNA)	**Biochemical:** High dose (71 %) Low dose (29 %) Controls (8 %) **Virological:** High dose (71 %) Low dose (36 %) Controls (0 %) **Complete:** High dose (50 %) Low dose (21 %) Complete (0 %)
Yuradaydin C et al. [61]	Non-randomized	39	IFN a-2a (9M) Vs. Lamivudine 100 mg/day vs. 2 months lamivudine followed by IFN a-2a + Lamivudine	12 months	At EOT: **Biochemical** (ALT) **Virological** (HDV RNA eradication) **Histological** **SVR** (6 months post-treatment)	Combination treatment was not superior to IFN monotherapy.
Yuradaydin C et al. [62]	Non-randomized	23	10 MU interferon alpha 2b, thrice weekly	24 months	At EOT and 6 months post-treatment: **Biochemical** (ALT) **Virological** (HDV RNA eradication) **Histological**	**Biochemical (ALT):** 47 % had a biochemical response but only 2 (13 %) had normal ALT after follow-up **Virological**: observed in 40 % patients at EOT and 13.3 % at follow-up. **Histological**: observed in 53 % patients 2 years of treatment does not increase SVR over 1 year treatment.
Castelnau C et al. [63]	Non-randomized	14	PEG-IFN alpha-2b (1.5 μg/kg/week)	12 months	**SVR**	**Virological:** EOT: 8 patients (57 %) SVR: 6 patients (43 %) **Biochemical:** 8 patients (57 %) were responders at EOT
Niro GA et al. [48]	Non-randomized	38	PEG-IFN alpha-2b monotherapy (72 weeks) PEG-IFN alpha 2b + RBV (48 weeks) followed by PEG-IFN monotherapy (24 weeks)	72 weeks		**Virological:** PEG IFN: (19 %) PEG IFN + RBV: (9 %) **Biochemical:** PEG IFN: (37.5 %) PEG IFN + RBV: (41 %)
Wedemeyer et al. [42]	RCT	90	PegIFN alfa-2a + adefovir vs. PegIFN alfa-2a + placebo vs. Adefovir alone	48 weeks	**Clearance of HDV** **RNA** **Normalization of ALT** **Decline in HBsAg** **levels**	**EOT:** HDV RNA was negative in 23 % in the first group, 24 % in the second, and none in the third **SVR:** 28 % in the 1st and 2nd groups and none in the 3rd group. A decline in HBsAg levels of more than 1 log(10) IU /mL observed in 10/31 patients in the first group, 2/29 in the second, and none in the third

HDV may relapse if HBsAg remains positive [40]. In a long ago trial of IFN-alfa-2a therapy that compared no treatment to high-dose (9 million units) and low-dose (3 million units) IFN, given 3 times a week for 48 weeks, 50 % of the high-dose group had a complete response defined by biochemical response (normalization of ALT) in addition to virological response (negative HDV levels at the end of the treatment) , compared to no complete response in any of those in the low-dose or no-treatment groups. Although sustained clearance of HDV RNA was not maintained in the high-dose group following cessation of therapy, a long-term follow-up study (up to 12 years) demonstrated significantly improved survival and liver histology [45]. More recently, Peg-IFN has been increasingly used because of its longer half-life, which allows once weekly dosing. Although no head-to-head comparison trials have been carried out, two major reviews have not been able to definitively show that either type of IFN therapy is superior to the other. Although one recent systematic review of randomized trials found that 1 year of high-dose interferon-alpha monotherapy achieved higher levels of undetectable HDV RNA and normalization of ALT at the end of treatment when compared with Peg-IFN-alpha monotherapy (RR 4.14; 95 % CI 1.00–17.14), levels of HDV RNA suppression 24 weeks after the end of therapy were not significantly different [46]. Another systematic review comparing standard and Peg-IFN-alpha found pooled sustained virologic response rates of 19 and 29 % of patients, respectively [47]. However, the investigators were unable to reach definitive conclusions on superiority due to the variation in trial designs involving standard IFN and the small number of patients involved. In other studies, the combination of Peg-IFN with ribavirin or with adefovir has been compared to treatment with Peg-IFN alone [42, 48]. The combinations yielded no difference in SVR rate, although the combination of Peg-IFN and adefovir resulted in increased decline in HBsAg levels. Extending the duration of Peg-IFN therapy has shown no effect on response rate [49]. In a study from Turkey, 32 % of patients with advanced chronic liver disease achieved virological response with Peg-IFN treatment and there was no significant difference in treatment response between patients with advanced and non-advanced chronic liver disease [50]. Acute HDV hepatitis has been more challenging to treat. Patients with fulminant hepatitis have had no response to IFN-alpha therapy, and liver transplant is the only option for fulminant hepatic failure resulting from coinfection or superinfection with HDV [51].

## Future Treatment

There are several current areas of research that may ultimately lead to important HDV treatment advances. Prenylation of the last four amino acids on the large-HDAg (the so-called CXXX box) is important for the interaction of HDV antigen with HBsAg [43, 52, 53]. Many small-molecule inhibitors of protein farnesyltransferase (which plays a role in prenylation) have been developed. These molecules compete with CXXX peptide substrates or with farnesyl diphosphate [43]. Both in vitro and in vivo studies have demonstrated that inhibition of prenylation results in clearance of HDV infection. Currently, the National Institutes of Health is enrolling HDV patients in a double-blinded, randomized, placebo-controlled trial to investigate the effectiveness of 4 weeks of treatment with the prenylation inhibitor lonafarnib, an oral farnesyltransferase inhibitor, with post-therapy follow up for a total of 6 months to assess for a sustained virological response (http://www.clinicaltrials.gov/ct2/show/NCT01495585?term=lonafarnib&rank=11).

Myclurdex, an HBV entry inhibitor, may also hinder the establishment of HDV infection by breaking the cycle of hepatocyte infection and possibly re-infection [54]. REP 9AC is a nucleic acid-based amphipathic polymer (NAP) which inhibits the release of HBsAg from infected hepatocytes. REP 9AC is currently being evaluated in patients with chronic HBV in a proof-of-concept clinical trial. Interim data showed that seven out of eight patients treated with REP 9AC cleared HBsAg or had only residual levels [55, 56]. Clearance of HBsAg and development of anti-HBs occurred as early as 7 days and no later than 32 weeks. Since HDV requires HBsAg for complete replication and transmission, REP 9AC might ultimately become an important new treatment tool.

New research on RNA interference (RNAi)-based therapies shows that they may also have the potential to treat HBV/HDV coinfection. In recent proof-of-concept studies, it was shown that, in transient and transgenic mouse models, coinjection of a hepatocyte-targeted, N-acetylgalactosamine-conjugated melittin-like peptide (NAG-MLP) with a liver-tropic cholesterol-conjugated siRNA (chol-siRNA) targeting conserved HBV sequences yielded multilog repression of viral RNA, proteins, and viral DNA [57]. With production of HBsAg blocked via RNA interference, HDV viral release might be decreased or prevented, potentially allowing immune control to overtake viral replication and effect viral clearance [58–60]. Finally, in the same way that we can currently use changes in levels of cytokines such as IP10 to monitor patients’ responses to standard interferon therapy, we may in future be able to map cytokine levels in order to monitor responses to new immunotherapy agents used as treatments for HBV and HDV, such as lambda interferon and TLR7 agonists.

## Conclusion

HDV infection is far from being a disappearing disease. Studies have shown increased prevalence even in developed countries, including the US, Australia, and some countries in Europe, and very high prevalence in endemic regions, despite the implementation of widespread HBV vaccination. Immigrants from endemic countries have been shown to have increased risk. Recent studies provide increasing evidence that sexual transmission may be an important factor in the spread of HDV infection. Based on the totality of evidence showing increased disease progression and substantially increased risk of cirrhosis in CHB patients who are also infected with HDV, and the current studies showing higher than expected prevalence, it is time to call for HDV screening of all CHB patients. HDV viral load detection and measurement should be considered in all patients whether or not they are anti-HDV-positive. With universal screening of CHB patients for HDV, earlier diagnosis and consideration of treatment would be possible. Current treatment of HDV is IFN-based therapy with or without HBV antivirals, but current research indicates the possibility that prenylation inhibitors, entry inhibitors, HBsAg release inhibitors, or the other therapies currently in the pipeline discussed here may provide more effective therapy in the future. In addition, universal screening would serve the important public health goal of allowing patients to be educated on their status and on the need for HDV-negative patients to protect themselves against superinfection, and for HDV-infected patients to protect against transmission to others. Further studies and global awareness of HDV infection are needed.

## Abbreviations

BEA: Baseline Event-Anticipation
CHB: cChronic hepatitis B
chol-siRNA: cCholesterol-conjugated siRNA
HBsAg: hHepatitis B surface antigen
HBV: hHepatitis B virus
HCC: hHepatocellular carcinoma
HDV: Hepatitis Delta virus
NAG-MLP: N-acetylgalactosamine-conjugated melittin-like peptide
NAP: nNucleic acid-based amphipathic polymer
Peg-IFN: pPegylated interferon
qRT-PCR: qQuantitative reverse transcription-polymerase chain reaction
RNAi: RNA interference
